# Retooling Laser Speckle Contrast Analysis Algorithm to Enhance Non-Invasive High Resolution Laser Speckle Functional Imaging of Cutaneous Microcirculation

**DOI:** 10.1038/srep41048

**Published:** 2017-01-20

**Authors:** Surya C. Gnyawali, Kevin Blum, Durba Pal, Subhadip Ghatak, Savita Khanna, Sashwati Roy, Chandan K. Sen

**Affiliations:** 1 Center for Regenerative Medicine & Cell-Based Therapies, Department of Surgery, Davis Heart and Lung Research Institute, The Ohio State University Wexner Medical Center, Columbus, OH 43210, USA

## Abstract

Cutaneous microvasculopathy complicates wound healing. Functional assessment of gated individual dermal microvessels is therefore of outstanding interest. Functional performance of laser speckle contrast imaging (LSCI) systems is compromised by motion artefacts. To address such weakness, post-processing of stacked images is reported. We report the first post-processing of binary raw data from a high-resolution LSCI camera. Sharp images of low-flowing microvessels were enabled by introducing inverse variance in conjunction with speckle contrast in Matlab-based program code. Extended moving window averaging enhanced signal-to-noise ratio. Functional quantitative study of blood flow kinetics was performed on single gated microvessels using a free hand tool. Based on detection of flow in low-flow microvessels, a new sharp contrast image was derived. Thus, this work presents the first distinct image with quantitative microperfusion data from gated human foot microvasculature. This versatile platform is applicable to study a wide range of tissue systems including fine vascular network in murine brain without craniotomy as well as that in the murine dorsal skin. Importantly, the algorithm reported herein is hardware agnostic and is capable of post-processing binary raw data from any camera source to improve the sensitivity of functional flow data above and beyond standard limits of the optical system.

Microcirculation, usually involving small arteries (<150 μm in diameter), arterioles, capillaries, and venules^1^,^2^, plays a critical role in physiological processes such as tissue oxygenation and nutritional exchange. Cutaneous vascular dysfunction is frequently associated with prevalent diseases condition such as type 2 diabetes, peripheral vascular disease (PVD), atherosclerotic coronary artery disease, obesity, heart failure, Alzheimer’s, schizophrenia and hypertension, among others^3^,^4^,^5^,^6^,^7^,^8^,^9^. Peripheral vascular dysfunction compromises dermal wound healing outcomes^10^,^11^. Quantification of dynamic blood flow, angiogenesis, and vessel density are critical for monitoring the progression of wound healing^12^,^13^,^14^. Although high resolution vascular network mapping is possible using imaging modalities such as computed tomography (CT), these approaches require injection of contrast agents^15^ and pose disadvantages such as radiation exposure^16^. Existing non-invasive methodologies including optical microscopy and laser Doppler-based imaging are inadequate to study blood flow at the resolution of gated single microvessels^17^,^18^. Moreover, these imaging modalities are motion-sensitive and thus may introduce variations by averaging the perfusion over an area^19^,^20^. Novel approaches to correct such limitations enabling high-resolution gated quantitation of microcirculatory blood flow is therefore of paramount interest.

Laser speckle contrast imaging (LSCI) enables non-contact, real-time, and non-invasive monitoring of changes in cutaneous blood flow^21^. This technique involves imaging of time-integrated speckle patterns generated by low-power laser irradiation employing a high spatiotemporal resolution charged coupled device (CCD) camera^22^,^23^,^24^,^25^. LSCI has several advantages over existing methods including simultaneously high spatial and temporal resolution, ease of implementation, and relatively low-cost of implementation^26^,^27^. However, functional performance of LSCI systems is limited by motion artefacts. To address this barrier, post-processing of stacked images is reported^28^,^29^,^30^. Such approach relies on averaging information gathered from multiple sequential frames. As a result, this approach is limited by the quality of images produced by traditional LSCI. Not limited by what is visualized in standard images, we report the first post-processing of binary raw data from a high-resolution LSI camera. In this adopted approach, final performance does not rely on the quality of traditional images. Instead, the raw binary data is subjected to noise correction, including correction of motion artefact as well as variance, which affects low-flow areas of interest. Based on such sensitivity, a new image with improved contrast is derived. In this work, we retooled the laser speckle contrast analysis (RT-LASCA) algorithm by writing a code to enable visualization and quantification of microcirculatory blood flow through a gated microvessel of interest.

## Results

### Graphical user interface to enhance visualization of dermal skin microvasculature and mapping of vascular anatomy

**Figure 1:**
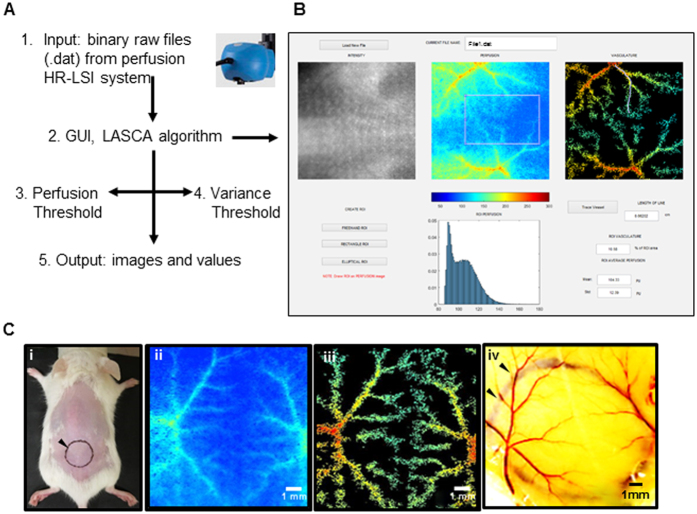
Flow chart to develop a graphical user interface (GUI) and mapping of vascular anatomy using macrophotography and retooling of perfusion based microvasculature. (**A**) Matlab platform was used to develop GUI and programming to read binary videos from the high resolution laser speckle imaging (HR-LSCI) system, use of RT-LASCA algorithm to compute speckle contrast, threshold on perfusion and variance with computed values and vascular images are shown. (**B**) GUI developed to run the binary videos, perform calculation and output images and data values are shown. (**C**) i- Digital image of a representative mouse with circular region of interest, ii- laser speckle perfusion image of the same ROI, iii-RT-LASCA image using the developed GUI. iv- Digital macrophotograph showing actual vasculature of the skin. The black arrowheads show the India ink used for marking the ROI.

A high resolution laser speckle contrast imaging (HR-LSCI) system was utilized to acquire structural and functional data from cutaneous microcirculation. The binary raw data files from the laser speckle imager (.dat files) was used as input for the RT-LASCA software and then processed to generate the microvascular map (Fig. 1A). The graphic user interface (GUI) included the gray-scale intensity image, the perfusion image, and the retooled microvasculature image as the output of LSCI binary raw data. The code (see ***Appendix A***) also included features that enabled drawing of free hand, rectangular and elliptical shaped region of interests (ROIs) that may be used to gate specific microvascular structures. Measurement of vascular length was obtained using free-hand tracing from GUI (Fig. 1B). The GUI also computed mean perfusion of a single microvessel, as well as percentage of perfused vasculature within the selected ROI (Fig. 1B). Images from RT-LASCA developed in this study showed microvessels consistent with cutaneous microvascular network as depicted in macrophotographic images of the murine skin (Fig. 1C). Reproducibility of the RT-LASCA images was verified by repeated acquisition of HR-LSCI images from the same ROI on murine dorsal skin over different time periods (Suppl. Fig. S1A). Branching of microvessels were visualized from post-processed RT-LASCA images (Suppl. Fig. S1B,C). The re-tooled software was capable of enumerating branching pattern of microvessels (Suppl. Fig. S1D).

### Structural and functional assessment of murine cutaneous microvasculature

**Figure 2:**
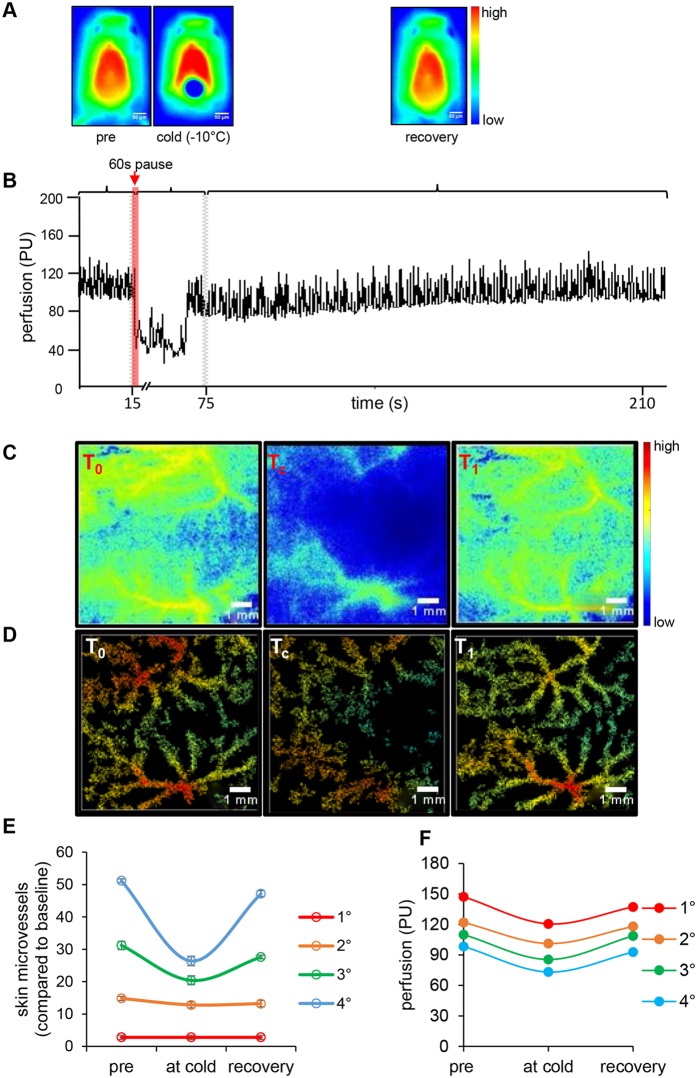
Structural and functional assessment of murine cutaneous microvasculature. (**A**) Thermal images of mice representing pre-, cold, and recovery time points. (**B**) Representative perfusion plot of cold treatment mouse. (**C**) Representative HR-LSCI perfusion images of mouse skin baseline, at the time of cold stimulation and recovery respectively, (**D**) corresponding RT-LASCA images. (**E**) Line graph representing the number of perfused microvessels as the ratio 1°:2°:3°:4 of number perfused vessels were plotted. (**F**) Line graph indicating changes in the perfusion at the three temperature changing time points indicated in the perfusion plot. Primary (1°), secondary (2°), tertiary (3°) and quaternary (4°) blood vessels. T_0_ = baseline, T_c_ = cold stimulation for 60 s. T_1_ = recovery. Data = mean ± SD, n = 4.

Cold-induced vasoconstriction was performed to study the structural and functional aspects of murine cutaneous microvasculature. Application of cold treatment on the dorsal skin of mice (Fig. 2A) limited perfusion by 60%, a response that returned to baseline after 210 seconds of treatment (Fig. 2B). The retooled LASCA software, developed in this study, was used to process the binary raw data files (Fig. 2C). The RT-LASCA images helped visualize fine cutaneous vascular network and assess blood flow in cold-affected individual gated vessels (Fig. 2D). The number of perfused vessels and their branches were quantified and plotted graphically (Fig. 2E). Perfusion data showed 11–27% reduction of blood flow in tertiary and quaternary vessels while flow in primary and secondary vessels remained unaffected (Fig. 2F). Changes in blood flow in response to different thermal conditions were also investigated. The perfusion of tertiary and quaternary microvessels was more sensitive to thermal variations when compared to flow in primary and secondary microvessels (Suppl. Fig. S2A–D).

### RT-LASCA detected microvessels in murine brain without craniotomy

**Figure 3:**
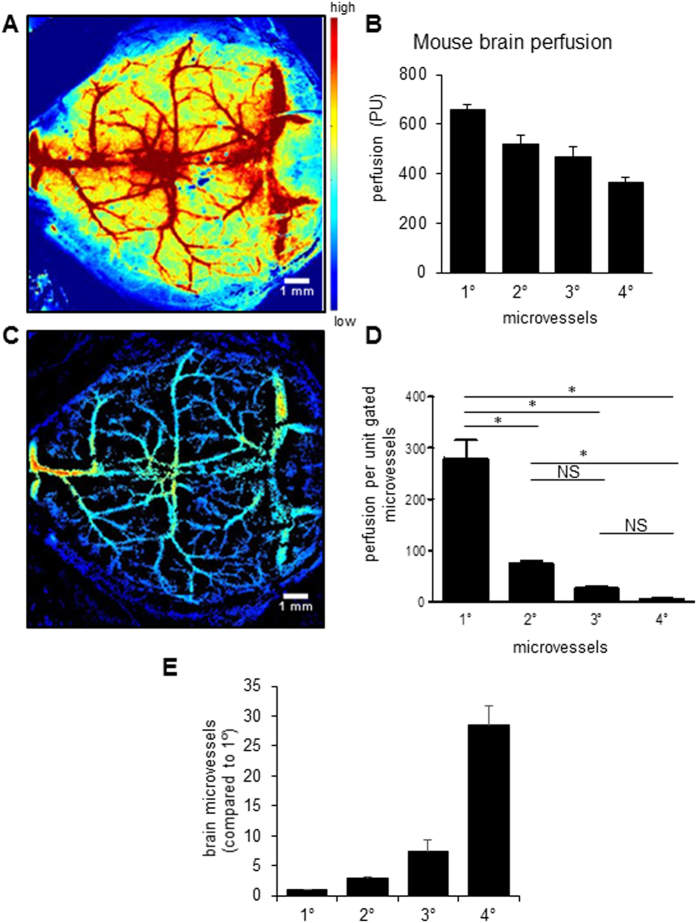
RT-LASCA detects microvessels in murine brain without craniotomy. (**A**) Representative LSCI perfusion image of mouse brain, (**B**) corresponding RT-LASCA image, (**C**) bar graph showing the perfusion of brain microvasculature, (**D**) graph indicating number of perfused microvessels, (**E**) Thresholded image in (**B**) to enable easier counting of the number of primary (1°), secondary (2°), tertiary (3°) and quaternary (4°) blood vessels. Data = mean ± SD, n = 3.

The RT LASCA algorithm was extended to study the cerebral vascular network in murine brain without opening the cranium. As shown in Fig. 3A, the HR-LSCI generated a high resolution perfusion map of the murine brain that was quantified based on the signal to noise ratio of the ROI (Fig. 3B). The RT-LASCA image generated based on LSCI binary raw data showed fine vascular network of the brain (Fig. 3C). Quantification of gated microvessels revealed a difference in cerebral microvascular blood flow among the vascular network that was previously not perceivable from LSCI images (Fig. 3D). Furthermore, this tool helped quantify the microvasculature of the heavily packed cerebral vascular network revealing the abundance of tertiary and quaternary microvessels compare to the primary and secondary vessels in the murine cerebral cortex (Fig. 3E).

### RT-LASCA detected cutaneous micro perfusion in human feet

**Figure 4:**
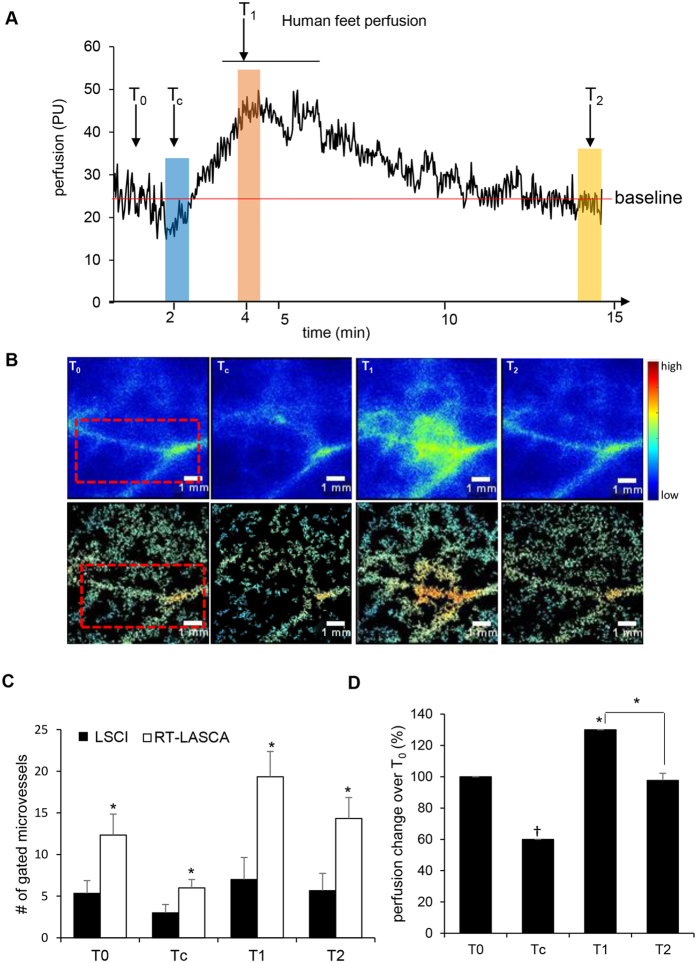
RT-LASCA detects micro perfusion in human feet. (**A**) Real-time perfusion graph showing the changes in vascular perfusion over time in human feet during cold treatment. (**B**) Representative LSCI perfusion images of human feet skin baseline, at the time of cold stimulation, reactive hyperemia and recovery respectively, (upper panel) corresponding RTLS images (lower panel). (**C**) Bar graph showing number of gated perfused microvessels during cold stimulation. Data represent number of gated perfused microvessels compared between LSCI and RT-LASCA images. (**D**) Bar graph showing change in perfusion of gated microvessels over baseline (T_0_). Scale bar = 1 mm. Data = mean ± SD, ^†^p = 0.002, cold stimulation vs normal, *p = 0.02, hyperemia vs normal, ns = non-significant, recovery vs normal, n = 6. T_0_ = baseline, T_c_ = cold stimulation for 1 min. T_1_ = reactive hyperemia, T_2_ = recovery.

Unlike murine skin, human skin has a thicker epidermis that absorbs more frequency thereby hindering perfusion measurement. We evaluate the ability of the RT-LASCA to detect human cutaneous microcirculatory network from the LSCI images. To transiently arrest microcirculation, the skin was subjected to a brief cold treatment for 1 min. Cold induced vasoconstriction (from baseline 30 PU to 15 PU) was followed by expected reactive hyperemia (45PU) in human feet (Fig. 4A). The LSCI binary raw data were processed through RT-LASCA resulting in the vivid demonstration of reduced perfusion specifically in tertiary vessels following cold treatment (Fig. 4B). Quantification of gated microvessels based on LSCI images and RT-LASCA image showed significant difference at different temperature showcasing the sensitivity of RT-LASCA (Fig. 4C). The differences in perfusion at different temperature were plotted graphically (Fig. 4D).

## Discussions

Non-homogenous spatial distribution of the microvasculature and high temporal variability in perfusion present challenges in physiological measurement. Microvascular evaluation can be performed with both local perfusion measurements techniques such as laser doppler flowmetry (LDF) and imaging methods such as laser speckle contrast imaging (LSCI). LSCI provides a full-field image of microcirculatory blood flow in tissue systems^31^. Considering the spatial heterogeneity of microvascular blood flow in tissues, perfusion mapping is preferred over spot measurements. Furthermore, perfusion mapping is more reproducible and may cover larger areas compared to single-point methods^18^. LSCI is a non-contact and non-invasive technique that enables rapid real-time measurement of superficial perfusion with high reproducibility^21^,^32^. However, because LSCI is motion sensitive, motion artifacts may cause overshooting of the perfusion value making it difficult to visualize and quantify the corresponding microvessels. In this work, we retooled the LASCA algorithm to provide high resolution images of peripheral microvasculature by post-processing of LSCI binary raw data. Application of RT-LASCA, thus derived, in post-processing perfusion data resulted in the following major improvements: (1) dynamic changes in blood flow in peripheral gated microvessels can be objectively measured in human feet; (2) quantitation of the primary (1°), secondary (2°), tertiary (3°) and quaternary (4°) perfused vessels from gated human leg skin is possible; (3) enabled quantitative assessment of cerebro-cortical microcirculation through the intact murine skull. Overall, this work demonstrates that perfusion-based, rapid, non-contact, non-invasive peripheral microvessel imaging is feasible. This has broad clinical applications. Importantly, the spatial resolution and gating opportunities made available as a result of this work are unprecedented for any optical imaging platform applicable to human-scale outcomes studies.

The algorithm reported here was primarily designed to demonstrate feasibility, with constraints largely based on motion artifacts and related noise. Motion-free imaging, albeit desirable, is rarely achievable in a non-invasive setting to study human functional outcomes^33^,^34^,^35^. Advancements in laser optics have improved signal strength, resolution, and the frame rates^33^,^36^,^37^. Implementation of a large area reconstruction and smoothing would likely require that backscattered light signal be received by the imaging camera^13^,^36^. Even with these added acquisition and registration abilities, motion compensation is not trivial^34^. The current literature addresses this limitation by post-processing stacked LSCI images^28^,^29^,^30^. However, the importance of variance on the contrast data over time has not been considered in such approaches. This is a significant oversight considering the fact that variance of contrast is a major confounding factor especially in low-flow areas^38^. In such areas of low perfusion, the variance of the contrast is known to be random in space and time with a high average value. In contrast, in areas of high perfusion, variance is less of a confounding factor while still demonstrating randomness. A simple and practical step in circumventing this problem is to develop post-processing solutions to visualize poorly perfused microvessels *via* statistical and thresholding elimination of noise and unwanted signals. The inclusion of inverse variance (1/V) factor into our algorithm in conjunction with speckle contrast factor (σ_I_/<I>) addresses variance as a confounding factor. Thus, the current work offers a novel and powerful tool for non-invasive detection of superficial microvascular anatomy as well as functional blood flow that may markedly influence cutaneous or cerebro-cortical perfusion related complications.

Studies on cold-induced vasoconstriction in human feet demonstrated the capability of our algorithm to quantitate reduction in blood flow followed by reactive hyperemia in gated microvasculature. Interestingly, our comparative studies led to the observation that the expected reactive hyperemia following cold stimulation observed in humans was not observed in mice pointing towards a clear difference in human and mouse vascular responses to transient cold exposure. Generally speaking, clearly there are several parallels between human and murine microcirculation. As a result mice are commonly used to model human microcirculation. For example, both have comparable right atrial and mean systemic arterial pressures (5 mm and 100 mm Hg, respectively), and similar capillary diameter of 6 μm^39^. However, there are clear contrasts too that cannot be explained in terms of allometric scaling^39^. One major difference is explained by a remarkable five-fold difference in heart rate between the two species^39^. As a result, mean blood flow velocity is far higher in mice. Humans have a mean flow velocity of 11 cm/s^40^ whereas average murine blood flow velocity is approximately 18.2 cm/s^41^. In addition, murine skin is thin and loose compared to humans^42^. These differences may account for the lack of reactive hyperemia response in mice subjected to topical cooling as observed in this study.

Other non-invasive tools to study organ perfusion include magnetic resonance imaging (MRI). MRI has been used both by others^43^,^44^ and our laboratory^45^ to study tissue circulation. MRI is commonly used in the assessment of brain abnormalities, stroke, vascular and degenerative diseases^46^. However, perfusion MRI is an expensive and complex technique that unable to provide accurate visualization and quantification of microvessels because of limitations in resolution (117 μm compared to 10–20 μm of HR-LSCI)^47^,^48^. Thus compared to MRI imaging, RT-LASCA software perfusion images can be generated at a relatively low cost with higher accuracy. Indeed RT-LASCA enabled the first optical visualization of cerebro-cortical microcirculation through the intact skull. Thus, RT-LASCA is a promising alternative to complicated and expensive MR-based perfusion imaging^49^. Perfusion maps of speckle contrast (K) can be generated using temporal statistics that exhibit vessel contrast images corresponding to the perfusion images. Retooled images of vasculature exhibited higher spatial resolution than the corresponding perfusion images. Such higher resolution enabled improved visualization of microcirculation. During computation of the K maps, there is an inherent decrease in the spatial resolution^35^. To compensate, a 5 × 5 sized sliding window was used depending on the quality of acquired perfusion data. In deciding the size of sliding window, there is a mathematical tradeoff where a smaller window (3 × 3) limits the statistical analysis, and a larger window (7 × 7) limits spatial resolution^50^,^51^. The resolution difference in the K maps generated using spatial *versus* temporal statistics predisposes the need to use a sliding window operator to compute K^35^. Using Matlab programming platform, we report a powerful image processing tool to visualize and quantitatively assess peripheral microvascular function^31^. The ability to visualize 3° and 4° vessel branches simply by applying an algorithm to improve the resolution of existing images without the use of contrast dyes is unparalleled in existing methodologies. Most studies demonstrating such level of detail have primarily been performed using dye-contrast methods or variations of optical coherence tomography (OCT) to study retinal vasculature^52^. As it relates to the cutaneous microcirculation, however, limitations in spatial and optical resolution and the problem of high background noise complicate microvascular imaging^53^,^54^.

In summary, the aim of the study was to enable collection of functional blood flow data from gated individual microvessels beyond the standard optical limits of the corresponding CCD camera. Thus, the algorithm we report is applicable to post-processing of signal from any source independent of the manufacturer. Resolution of functional LSCI data depends on two major factors: (i) optical limits of the camera, and (ii) post-processing approach. Because this work is hardware agnostic, our focus has been on the latter. Thus, any improvement in hardware technology will only further enhance the capability of our algorithm as it relates to improving the resolution of functional data acquisition. The RT-LASCA algorithm presented in this work enabled gating of a single microvessel on superficial tissue and obtained maps of primary, secondary, tertiary, and quaternary blood vessels. Quantitative assessment of perfusion and determination of dimensions of each microvascular branch was enabled. Thus, this work presents a novel tool to assess peripheral microcirculation both in research as well as clinical settings.

## Materials and Methods

### Animal preparation

All animal procedures were approved by the Institutional Animal Care and Use Committee of The Ohio State University and performed in accordance with their relevant guidelines and regulations. The animal experiments were performed in accordance with the approved guidelines. Adult (>8 weeks old) male NOD.Cg-Prkdcscid Il2rgtm1Wjl/SzJ mice (Stock No: 005557/NSG mice, The Jackson Laboratory, Bar Harbor, ME, USA) were used in all experiments. Standard rodent chow (Harlan Laboratories, Indianapolis, IN, USA) and water *ad libitum* was provided as per standard protocol at the Ohio State University animal vivarium. During experimentation mice were anesthetized using isoflurane and medical air mixture through a nose cone and placed on a 37 °C warm surgical plate. The dorsal skin of the mouse was depilated 24 hours prior to the experiment.

### Laser Speckle Perfusion Imaging

Mice were anesthetized and placed on the warm plate maintained at 37 °C. After the respiration was stabilized, perfusion recordings were performed using a high resolution (20 μm) laser speckle camera (PeriCam PSI HR System, PeriMed, Sweden). Data were acquired from a 1.5 cm × 1.5 cm field of view using a 785 nm, 80 mW laser with a sampling rate of 60 Hz at a working distance of 10 cm. Relative perfusion units were averaged over a 10 s sampling period. After the completion of perfusion recordings mice were allowed to recover in the cages with food and water. From the real-time perfusion graphs, time-of-interest (TOI) was chosen to include lower peaks only to exclude the respiratory motion related artifacts. Average perfusion was calculated by using PimSoft v1.4 software (Perimed Inc., Jarfalla, Sweden).

### Software Development

Laser speckle contrast analysis (LASCA) utilizes the inherent properties of reflected light to gather information that is not visible to the naked eye. When coherent light reflects off a surface, the rebounded speckle pattern is inherently random in both space and time, creating a Gaussian distribution of speckle intensity, with a standard deviation equal to the average intensity^55^. However, when reflecting off a moving surface, such as moving red blood cells in the vasculature, the light is reflected more heavily away from that region in space in the direction of flow, creating a blur^35^,^56^. This blur is characterized by a lower standard deviation with respect to the intensity at that point. This blur is typically quantified as “speckle contrast” and is defined as:


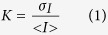


where K is the speckle contrast, ***σ***_I_ is the standard deviation of the speckle intensity, and <I> is the mean intensity around the point in space and/or time. Due to a relation between the speckle pattern and blur, there is a mathematical limit of K ≤ 1, and a lower K value represents more blur^55^. This contrast can be correlated to blood flow through the laser speckle contrast analysis (LASCA) algorithm, which in the case of the Perimed PSI system (Perimed-instruments Inc.) is defined as:


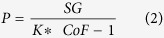


where SG is the signal gain of the system and CoF is the Coherence Factor of the system, both of which are set in the Perimed system. The resulting P value is the estimated perfusion of the area of interest in arbitrary “perfusion units” (PU).

The high resolution laser speckle imaging (HRLSCI) system was set to record speckle images at a rate of 15 frames per second, calculating intensity and variance with spatiotemporal averaging over a 5 × 5 pixel area and 5 frames. Corresponding binary raw data were used for further processing in Matlab program (Mathworks, Natick, MA). This sets the overall frame rate for the intensity and variance data at 3 frames per second, and the full imaging time to record the vasculature in the experiments was 15 s. As this method effectively increases the time of interest, it is expected for this to decrease the variability of the signal^51^. In order to reduce the effects on the mean of large sudden changes in perfusion readings, such as due to breathing motion, the median value of perfusion was chosen for each pixel.

The retooled LASCA algorithm (RT-LASCA) also utilizes the variance of the contrast data over time to determine the locations of vasculature. The variance of the contrast (V) was found to vary with respect to the underlying blood flow. In areas of low perfusion, the variance of the contrast was random in space and time with a high average value, while in areas of high perfusion, variance was lower, while still demonstrating randomness. Due to the low values of contrast variances which comprise the signal of interest (vasculature), and the large, exponentially decaying nature of the variances of the tissue signal, the contrast variances were inverted (1/V) to suppress the noise values into a smaller range and accentuate the range of signal values.

To locate the vasculature, two masks were created of likely vasculature locations, and then compared. The perfusion map was threshold by comparing to the local mean. Specifically, each pixel was compared to the mean of a 100 by 100 square matrix of pixels surrounding it. Pixels found to have a higher perfusion value than local mean were deemed as “pass” and assigned a value of 1, while the other pixels were assigned a “fail” value of 0. The size of the matrix was chosen to be larger than the blood vessels being examined, to increase the likelihood that vessel regions would be given a value of 1. This method was employed to increase the contrast in areas where the perfusion of the vessel was similar in magnitude to the perfusion of the surrounding tissue. The resulting binary matrix represented a “mask” of pixels that were likely to contain vasculature. A similar mask was then created using the inverse variance map.

The threshold masks were then filtered to remove speckle-related random noise. This was done by utilizing the fact that blood vessels are part of an interconnected vascular tree, and as such any pixels that are “pass” in the vasculature masks but are not connected to other “pass” pixels are likely statistical noise. Because statistical noise could also result in vasculature being labeled as “fail” pixels, and therefore break up the vascular tree, a filter size of 1000 interconnected pixels was experimentally determined as an appropriate cutoff to retain as much of the likely vasculature as possible. Any pixel that was “pass” (assigned a value of 1) in the mask, but was not shown to be interconnected in a set of at least 1000 “pass” pixels, was updated to a “fail” pixel (assigned a value of 0).

Finally, the inverse variance and perfusion masks were compared, and areas which were shown to be positive in both masks were retained and assumed to be vasculature. This vasculature map was then recolored using the original perfusion map data to show the perfusion within this vasculature. During development of GUI, code was written based on Matlab platform (Suppl. Fig. S2). In order to calculate vascular density, code was written in Matlab to allow the tracing of vasculature by hand. These tracings were then converted into lengths of vessels using the Pythagorean Theorem between points along the tracing:


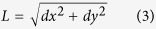


where L is length of vessels and *dx* and *dy* are tracings along x- and y- coordinates respectively. The lengths calculated by the Pythagorean Theorem were then summed and divided by the area of the defined region of interest (ROI) to find the vascular density, in cm^−1^. The tracing feature was used to count the number of vessels and find the total length of vessels within the ROI. Specific details of all coding steps can be found in the source code, provided as Supplemental Material.

### Mapping of vascular anatomy using microphotography and retooling of perfusion based microvasculature

A circular region was marked on the dorsal skin of NSG mice as region of interest to validate the vascular pattern and morphology. Perfusion imaging was performed on the marked region. To validate the recording, the skin was folded out *via* standard surgical procedure.

### Cold mediated restricted blood perfusion and thermal mapping in mice

Mice were anesthetized and baseline perfusion was measured. A cold treatment was applied at ROI for 60 s, and then thermal recording was continued until recovery using thermal imaging (FLIR thermovision, Canada). Thermal and perfusion images were acquired at baseline (pre), cold, and recovery. All the binary files were processed by RT-LASCA software. Perfusion and number of microvessels were also computed. A line graph was plotted at three different time points of cold stimulation. Microvessels were categorized to primary (1°), secondary (2°), tertiary (3°) and quaternary (4°) blood vessels based on the perfusion.

### RT-LASCA detection of dynamic microvessels in mice brain

Mice were anesthetized and the scalp was opened surgically following standard surgical procedure. Following this, the brain was subjected to HR- LSCI perfusion imaging through the intact skull. Mice were allowed to recover post-imaging. Binary files were processed using RT-LASCA software to identify and quantify perfusion in 1°, 2°, 3° and 4° blood vessels. Perfusion per unit gated microvessels were computed.

### Cold mediated restricted blood perfusion in human feet

All human studies were approved by The Ohio State University’s (OSU) Institutional Review Board (IRB) and performed in accordance with their relevant guidelines and regulations. Patients gave their written informed consent prior to the experiment. Baseline perfusion measurement was performed by high resolution laser speckle imaging. Cold treatment was applied on the skin surface at ROI for 60 s and the recording was continued until the perfusion level reverts back to normal. Selective time of interests (TOIs) were used to derive raw binary data files to process in the proposed software. Gated microvessels were quantified and perfusion was measured.

## Additional Information

**How to cite this article**: Gnyawali, S. C. *et al*. Retooling Laser Speckle Contrast Analysis Algorithm to Enhance Non-Invasive High Resolution Laser Speckle Functional Imaging of Cutaneous Microcirculation. *Sci. Rep.* **7**, 41048; doi: 10.1038/srep41048 (2017).

**Publisher's note:** Springer Nature remains neutral with regard to jurisdictional claims in published maps and institutional affiliations.

## Supplementary Material

Supplementary Information
