# Supplementary material for: Retooling Laser Speckle Contrast Analysis Algorithm to Enhance Non-Invasive High Resolution Laser Speckle Functional Imaging of Cutaneous Microcirculation
Source: Sci Rep. 2017 Jan 20;7:41048. doi: 10.1038/srep41048 (PMC5247692; doi:10.1038/srep41048)
Supplement: Supplementary Information [file srep41048-s1.pdf]

Supplementary Information

Retooling Laser Speckle Contrast Analysis Algorithm to Enhance Non-Invasive High Resolution Laser Speckle Functional Imaging of Cutaneous Microcirculation

Surya C Gnyawali[1‡], Kevin Blum[1‡], Durba Pal[1], Subhadip Ghatak[1],
Savita Khanna[1], Sashwati Roy[1], Chandan K Sen[1*]

1 Center for Regenerative Medicine & Cell-Based Therapies,
Department of Surgery, Davis Heart and Lung Research Institute,
The Ohio State University Wexner Medical Center, Columbus, OH 43210, U.S.A.

‡ These authors contributed equally to this work.

***Corresponding Author:** Chandan K. Sen
Professor & Director
Center for Regenerative Medicine & Cell-Based Therapies
The Ohio State University Wexner Medical Center,
473 West 12th Ave, Columbus, OH 43210.
Tel.: 614-247-7658; Fax: 614-247-7818
E-mail: chandan.sen@osumc.edu

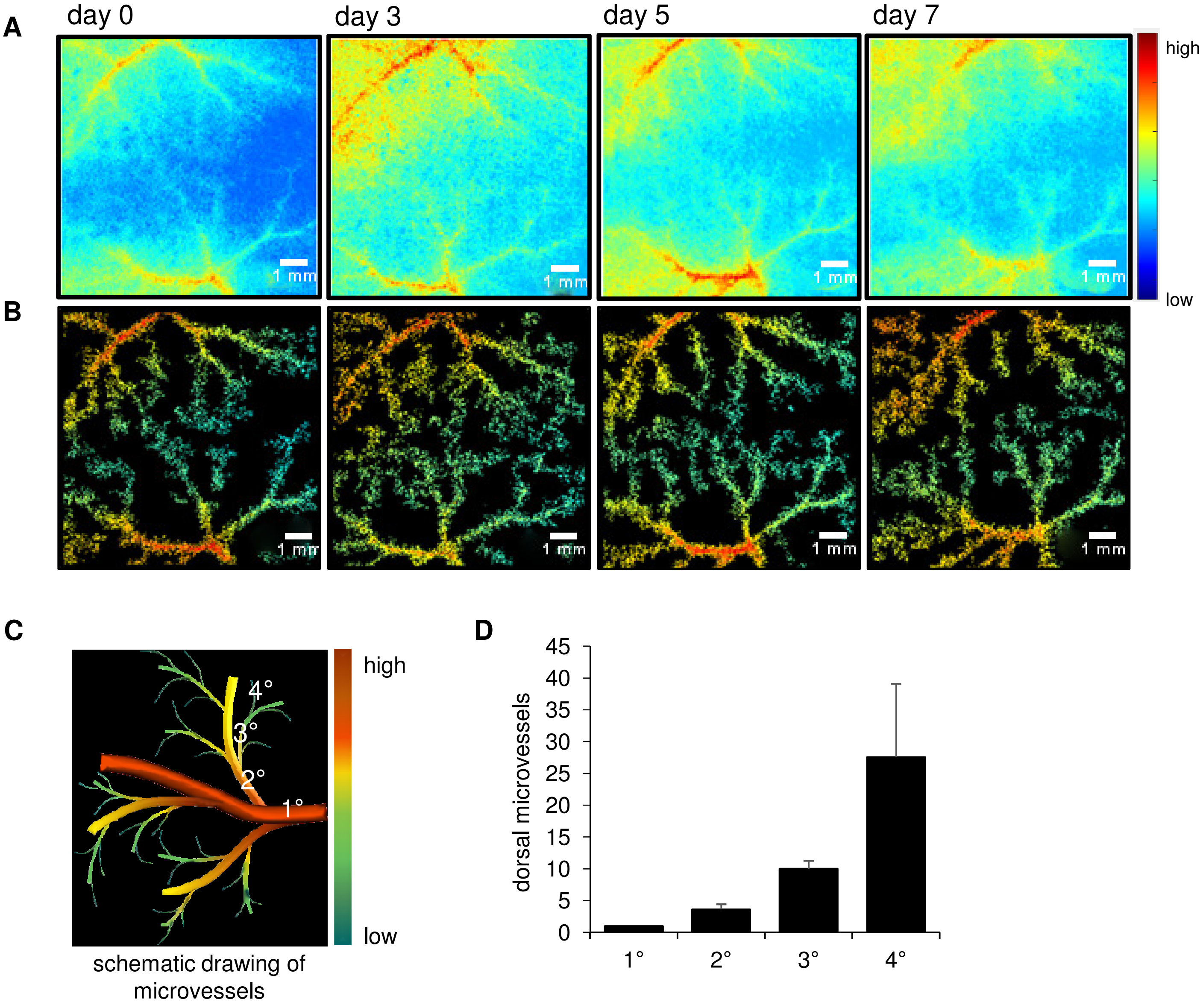

Figure S1. Reproducibility of RT-LASCA from laser speckle perfusion images (**A**) LSI perfusion maps, (**B**) RT-LASCA images for the same mouse for days 0, 3, 5, and 7. Color scale represents perfusion level. (**C**) Schematic drawing of microvessels. (**D**) The number of primary (1°), secondary (2°), tertiary (3°) and quaternary (4°) blood vessels were counted and the ratio 1° :2° :3° :4° of number perfused vessels were plotted. Data = mean ±SD, n = 3.

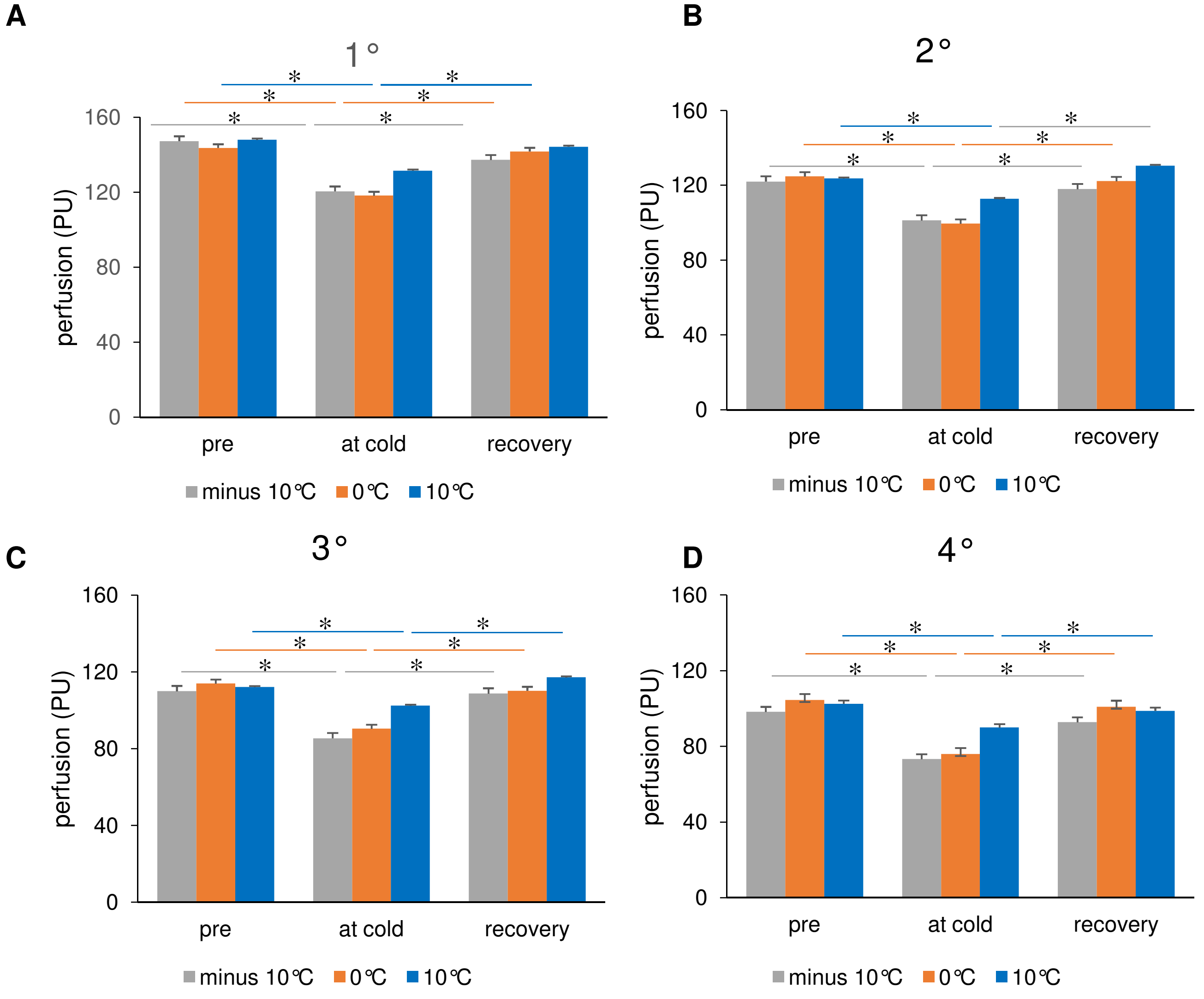

Figure S2. **RT-LASCA quantification of dynamic vascular changes induced by cold in mice.** Bar graph showing perfusion in microvessels, **(A)** primary, (B) secondary, (C) tertiary and (D) quaternary microvessels at three different temperature points (**-10**, **0** and **10** ° C). Data = mean ±SD, *p = 0.05, n = 4.

Appendix A

```
function varargout = BlumGUI(varargin)
global LocAvg;
global Pconnect;
global Kconnect;

%below are your filter values to adjust

%defines pixel radius for local averaging
%generally, smaller better for smaller vessels
LocAvg = 50; %100 is default

%defines pixel connectivity for perfusion data
%lower number = less restrictive
Pconnect = 10; %1000 is default

%defines pixel connectivity for K data
%lower number = less restrictive
Kconnect = 10; %1000 is default

%BLUMGUI MATLAB code for BlumGUI.fig
%      BLUMGUI, by itself, creates a new BLUMGUI or raises the existing
%      singleton*.
%
%      H = BLUMGUI returns the handle to a new BLUMGUI or the handle to
%      the existing singleton*.
%
%      BLUMGUI('CALLBACK',hObject,eventData,handles,...) calls the local
%      function named CALLBACK in BLUMGUI.M with the given input arguments.
%
%      BLUMGUI('Property','Value',...) creates a new BLUMGUI or raises the
%      existing singleton*.  Starting from the left, property value pairs are
%      applied to the GUI before BlumGUI_OpeningFcn gets called.  An
%      unrecognized property name or invalid value makes property application
%      stop.  All inputs are passed to BlumGUI_OpeningFcn via varargin.
%
%      *See GUI Options on GUIDE's Tools menu.  Choose "GUI allows only one
%      instance to run (singleton)".
%
% See also: GUIDE, GUIDATA, GUIHANDLES

% Edit the above text to modify the response to help BlumGUI

% Last Modified by GUIDE v2.5 20-Jul-2015 14:30:12

% Begin initialization code - DO NOT EDIT
gui_Singleton = 1;
gui_State = struct('gui_Name',       mfilename, ...
                   'gui_Singleton',  gui_Singleton, ...
                   'gui_OpeningFcn', @BlumGUI_OpeningFcn, ...
                   'gui_OutputFcn',  @BlumGUI_OutputFcn, ...
                   'gui_LayoutFcn',  [] , ...
                   'gui_Callback',   []);
if nargin && ischar(varargin{1})
    gui_State.gui_Callback = str2func(varargin{1});
end
```

```
if nargout
    [varargout{1:nargout}] = gui_mainfcn(gui_State, varargin{:});
else
    gui_mainfcn(gui_State, varargin{:});
end
% End initialization code - DO NOT EDIT


% --- Executes just before BlumGUI is made visible.
function BlumGUI_OpeningFcn(hObject, ~, handles, varargin)
% This function has no output args, see OutputFcn.
% hObject    handle to figure
% eventdata  reserved - to be defined in a future version of MATLAB
% handles    structure with handles and user data (see GUIDATA)
% varargin   command line arguments to BlumGUI (see VARARGIN)

% Choose default command line output for BlumGUI
handles.output = hObject;

% Update handles structure
guidata(hObject, handles);

% UIWAIT makes BlumGUI wait for user response (see UIRESUME)
% uiwait(handles.figure1);


% --- Outputs from this function are returned to the command line.
function varargout = BlumGUI_OutputFcn(~, ~, handles)
% varargout  cell array for returning output args (see VARARGOUT);
% hObject    handle to figure
% eventdata  reserved - to be defined in a future version of MATLAB
% handles    structure with handles and user data (see GUIDATA)

% Get default command line output from handles structure
varargout{1} = handles.output;


% --- Executes on button press in LOAD NEW FILE.
function pushbutton1_Callback(~, ~, handles)
% hObject    handle to pushbutton1 (see GCBO)
% eventdata  reserved - to be defined in a future version of MATLAB
% handles    structure with handles and user data (see GUIDATA)
global P;
global vessel;
global LocAvg;
global Pconnect;
global Kconnect;
set(handles.text11, 'string', 'Loading...Please Wait');
%open file, and import all information
filename = uigetfile('.dat', 'CHOOSE BINARY FILE'); %choose file
File = PIMSoftBinary; %set file name
File.OpenFile(filename); %open file
set(handles.edit1,'String', filename);
Frames = File.numberOfImages; %define number of frames in file
```

```
Height = File.imageHeight; %define height of image
Width = File.imageWidth; %define width of image
CoF = File.coherenceFactor; %define coherence factor of file
SG = File.signalGain; %define signal gain of factor
I = zeros(Height, Width, Frames); %blank matrix for intensity frames
Var = zeros(Height, Width, Frames); %blank matrix for variance frames

%load intensity and variance frames
for n = 1:Frames;
    I(:,:,n) = File.getDCFrame(n);
    Var(:,:,n) = File.getVarianceFrame(n);
end

K = sqrt(abs(Var))./I; %calculate K values for each frame
Iavg = mean(I, 3); %calculate temporal average intensity
Vavg = mean(abs(Var), 3); %calculate temporal average variance
Kavg = mean(K, 3); %calculate temporal average K values
Kstd = std(K, [], 3); %calculate K standard deviation
Kvar=Kstd.^2; %calculate K variance

C  = K*CoF; %combine K value with coherence factor
Pstack = SG*(1./C-1); %calculate perfusion values
Pmed = median(Pstack, 3); %find temporal median perfusion values
Pmed(isnan(Pmed)) = 0;
Pmed(Pmed>3000) = 0;
%convert values to 0-1 scale
Pmnew=(Pmed-min(min(Pmed)))/(max(max(Pmed))-min(min(Pmed)));

%invert K variance data
iV = 1./(Kvar+0.0001); %add 0.0001 to prevent Inf errors
V = iV/max(max(iV));

%create perfusion mask for perfusion data, and apply to perfusion data
mask = Pmnew<prctile(Pmnew(:), 5);
P = regionfill(Pmnew, mask);
%convert to 0-1 scale
P2 = P*(max(max(Pmed))-min(min(Pmed)))+min(min(Pmed));

%create local average filter
h = fspecial('average', LocAvg);
%apply local average filter to perfusion data
test = imfilter(P, h, 'replicate');
%connectivity mask for perfusion data
X = bwareaopen(test<P, Pconnect);
%apply local average filter to inverted K variance data
test2 = imfilter(V, h, 'replicate');
%connectivity filter for inverted K variance data
Y = bwareaopen(test2<V, Kconnect);

P = P2; %set scaled unfiltered perfusion data for display
cmap = [gray(1);jet(63)]; %create perfusion color map
vessel = X.*Y; %create vessel image
color = vessel*63.*P; %create colored vessel image

%display all images
```

```
axes(handles.axes1)
imshow(Iavg, []);
axes(handles.axes2)

%to set the min/max for perfusion image,
%change imshow(P, []) below to
imshow (P, [0, 250])
%imshow(P, []);%display perfusion map
p=get(handles.axes2,'position'); % save position
colorbar('southoutside');
set(gca,'position',p); % restore position
% you may have to tweak <p> (overlap)
colormap(handles.axes2, jet)
axes(handles.axes3)
colormap(handles.axes3, cmap)
imshow(color, []);
set(handles.text11, 'string', '    ');


% --- Executes on button press in FREEHAND ROI.
function pushbutton2_Callback(~, ~, handles)
% hObject    handle to pushbutton2 (see GCBO)
% eventdata  reserved - to be defined in a future version of MATLAB
% handles    structure with handles and user data (see GUIDATA)
global P;
global vessel;
global BW;
%create ROI
ROI = imfreehand(handles.axes2);

%wait for ROI to be determined, and define ROI
h = wait(ROI);
BW = createMask(ROI);

total = sum(sum(BW)); %total number of pixels in image
count = 1; %rolling counter
matrix = zeros(1,total); %list for all ROI pixels
BW2 = (2*BW-1).*P; %create perfusion ROI
%Find all perfusion values in ROI
for i = 1:size(BW2, 1);
for j = 1:size(BW2,2);
if BW2(i,j) > 0;
matrix(count) = BW2(i,j);
count = count+1;
else
end
end
end
%display histogram of ROI perfusion values
axes(handles.axes4)
histogram(matrix, 'Normalization', 'pdf')

%Calculate vascularity of ROI
Vasc = sum(sum(BW.*vessel))/total*100; %vascularity percentage
Vasc = round(Vasc*10^(2))/(10^(2)); %Round to hundredths place
set(handles.edit2,'String', Vasc); %display vascularity
```

```
AVG = mean(matrix); %average perfusion value in ROI
AVG = round(AVG*10^(2))/(10^(2)); %round to hundreths place
set(handles.edit3,'String', AVG); %display average
STD = std(matrix); %standard deviation of ROI perfusion
STD = round(STD*10^(2))/(10^(2)); %round to hundreths
set(handles.edit4,'String', STD); %display standard deviation
 % --- Executes on button press in ELLIPTICAL ROI.
function pushbutton4_Callback(~, ~, handles)
% hObject    handle to pushbutton4 (see GCBO)
% eventdata  reserved - to be defined in a future version of MATLAB
% handles    structure with handles and user data (see GUIDATA)
global P;
global vessel;
global BW;
 ROI = imellipse(handles.axes2);
 %wait for ROI to be determined, and define ROI
h = wait(ROI);
BW = createMask(ROI);

total = sum(sum(BW)); %total number of pixels in image
count = 1; %rolling counter
matrix = zeros(1,total); %list for all ROI pixels
BW2 = (2*BW-1).*P; %create perfusion ROI
%Find all perfusion values in ROI
for i = 1:size(BW2, 1);
for j = 1:size(BW2,2);
if BW2(i,j) > 0;
matrix(count) = BW2(i,j);
count = count+1;
else
end
end
end
%display histogram of ROI perfusion values
axes(handles.axes4)
histogram(matrix, 'Normalization', 'pdf')
 %Calculate vascularity of ROI
Vasc = sum(sum(BW.*vessel))/total*100; %vascularity percentage
Vasc = round(Vasc*10^(2))/(10^(2)); %Round to hundredths place
set(handles.edit2,'String', Vasc); %display vascularity
AVG = mean(matrix); %average perfusion value in ROI
AVG = round(AVG*10^(2))/(10^(2)); %round to hundreths place
set(handles.edit3,'String', AVG); %display average
STD = std(matrix); %standard deviation of ROI perfusion
STD = round(STD*10^(2))/(10^(2)); %round to hundreths
set(handles.edit4,'String', STD); %display standard deviation
 function edit1_Callback(~, ~, ~)
% hObject    handle to edit1 (see GCBO)
% eventdata  reserved - to be defined in a future version of MATLAB
% handles    structure with handles and user data (see GUIDATA)

% Hints: get(hObject,'String') returns contents of edit1 as text
%        str2double(get(hObject,'String')) returns contents of edit1 as a double

% --- Executes during object creation, after setting all properties.
function edit1_CreateFcn(hObject, ~, ~)
% hObject    handle to edit1 (see GCBO)
```

```
% eventdats  reserved - to be defined in a future version of MATLAB
% handles    empty - handles not created until after all CreateFcns called

% Hint: edit controls usually have a white background on Windows.
%       See ISPC and COMPUTER.
if ispc && isequal(get(hObject,'BackgroundColor'),
get(0,'defaultUicontrolBackgroundColor'))
    set(hObject,'BackgroundColor','white');
end



function edit2_Callback(~, ~, ~)
% hObject    handle to edit2 (see GCBO)
% eventdats  reserved - to be defined in a future version of MATLAB
% handles    structure with handles and user data (see GUIDATA)

% Hints: get(hObject,'String') returns contents of edit2 as text
%        str2double(get(hObject,'String')) returns contents of edit2 as a
double


% --- Executes during object creation, after setting all properties.
function edit2_CreateFcn(hObject, ~, ~)
% hObject    handle to edit2 (see GCBO)
% eventdata  reserved - to be defined in a future version of MATLAB
% handles    empty - handles not created until after all CreateFcns called

% Hint: edit controls usually have a white background on Windows.
%       See ISPC and COMPUTER.
if ispc && isequal(get(hObject,'BackgroundColor'),
get(0,'defaultUicontrolBackgroundColor'))
    set(hObject,'BackgroundColor','white');
end



function edit3_Callback(~, ~, ~)
% hObject    handle to edit3 (see GCBO)
% eventdata  reserved - to be defined in a future version of MATLAB
% handles    structure with handles and user data (see GUIDATA)

% Hints: get(hObject,'String') returns contents of edit3 as text
%        str2double(get(hObject,'String')) returns contents of edit3 as a
double


% --- Executes during object creation, after setting all properties.
function edit3_CreateFcn(hObject, ~, ~)
% hObject    handle to edit3 (see GCBO)
```

```
% eventdats  reserved - to be defined in a future version of MATLAB
% handles    empty - handles not created until after all CreateFcns called

% Hint: edit controls usually have a white background on Windows.
%       See ISPC and COMPUTER.
if ispc && isequal(get(hObject,'BackgroundColor'),
get(0,'defaultUicontrolBackgroundColor'))
    set(hObject,'BackgroundColor','white');
end

 function edit4_Callback(~, ~, ~)
% hObject    handle to edit4 (see GCBO)
% eventdata  reserved - to be defined in a future version of MATLAB
% handles    structure with handles and user data (see GUIDATA)

% Hints: get(hObject,'String') returns contents of edit4 as text
%        str2double(get(hObject,'String')) returns contents of edit4 as a double


% --- Executes during object creation, after setting all properties.
function edit4_CreateFcn(hObject, ~, handles)
% hObject    handle to edit4 (see GCBO)
% eventdata  reserved - to be defined in a future version of MATLAB
% handles    empty - handles not created until after all CreateFcns called

% Hint: edit controls usually have a white background on Windows.
%       See ISPC and COMPUTER.
if ispc && isequal(get(hObject,'BackgroundColor'),
get(0,'defaultUicontrolBackgroundColor'))
    set(hObject,'BackgroundColor','white');
end



function edit5_Callback(~, ~, ~)
% hObject    handle to edit5 (see GCBO)
% eventdata  reserved - to be defined in a future version of MATLAB
% handles    structure with handles and user data (see GUIDATA)

% Hints: get(hObject,'String') returns contents of edit5 as text
%        str2double(get(hObject,'String')) returns contents of edit5 as a double


% --- Executes during object creation, after setting all properties.
function edit5_CreateFcn(hObject, ~, ~)
% hObject    handle to edit5 (see GCBO)
% eventdata  reserved - to be defined in a future version of MATLAB
% handles    empty - handles not created until after all CreateFcns called

% Hint: edit controls usually have a white background on Windows.
%       See ISPC and COMPUTER.
if ispc && isequal(get(hObject,'BackgroundColor'),
get(0,'defaultUicontrolBackgroundColor'))
    set(hObject,'BackgroundColor','white');
```

```
end


% --- Executes on button press in Line Trace.
function pushbutton5_Callback(~, ~, handles)
% hObject    handle to pushbutton5 (see GCBO)
% eventdats  reserved - to be defined in a future version of MATLAB
% handles    structure with handles and user data (see GUIDATA)
ROI = imfreehand(handles.axes3, 'Closed', false);

%wait for ROI to be determined, and define ROI
h = wait(ROI);
Line = ROI.getPosition;
%Find length of drawn line
L = 0; %starting length equal to 0
%for each vertex of the line, calculate its 2D distance from
previous
%vertex and add to running total
for i = 1:(size(Line,1)-1)
    L = L + sqrt((Line(i,1)-Line(i+1,1))^2+(Line(i,2)-
Line(i+1,2))^2);
end
L = L*0.002; %multiply by 0.002 to convert to cm
set(handles.edit5,'String', L); %display length of line
```
